# Engaging Older Adults With Neurocognitive Disorders in Digital Health Technologies: Scoping Review

**DOI:** 10.2196/70157

**Published:** 2026-07-17

**Authors:** Sié Mathieu Aymar Romaric Da, Maxime Sasseville, Marie-Soleil Hardy, Idrissa Beogo, Amédé Gogovor, Samira Amil, Achille R Yameogo, Florian Naye, Farzaneh Yousefi, Frédéric Bergeron, Anik Giguère, Annie LeBlanc, James Plaisimond, Carole Rivard-Lacroix, Marie-Pierre Gagnon, Chloé Cachinho

**Affiliations:** 1 Faculty of Nursing Sciences, Université Laval,, Pavillon Ferdinand-Vandry, local 1426, 1050, Medicine Avenue, Quebec, QC, G1V 0A6, Canada; 2 VITAM, Research Center for Sustainable Health, Integrated University Health and Social Services Center (CIUSSS) of the Capitale-Nationale, Laval University, Québec, QC, Canada; 3 School of Nursing, University of Ottawa, Ottawa, ON, Canada; 4 Faculty of Medicine, Université Laval, Québec, QC, Canada; 5 School of Nutrition, Université Laval, Québec, QC, Canada; 6 Faculty of Health Sciences, Université de Sherbrooke, Sherbrooke, QC, Canada; 7 Library – Consulting Services Directorate, Université Laval, Québec, QC, Canada; 8 Institute for Aging and Social Participation of Seniors, Université Laval, Québec, QC, Canada

**Keywords:** engagement, older adults, neurocognitive disorders, digital health technologies, eHealth

## Abstract

**Background:**

Population aging is associated with a growing prevalence of neurocognitive disorders among adults aged 65 years and older. Digital health technologies offer promising opportunities to support cognitive health and well-being in this population. However, their effectiveness largely depends on users’ level of engagement. Despite the recognized importance of engagement in digital health, limited evidence exists on how engagement is conceptualized, measured, and related to intervention outcomes among older adults living with neurocognitive disorders.

**Objective:**

This scoping review aimed to describe how engagement with digital health technologies among older adults with neurocognitive disorders is conceptualized and measured, examine the relationship between engagement and the effectiveness of digital health interventions, and identify factors that facilitate or hinder engagement.

**Methods:**

A scoping review was conducted following the Joanna Briggs Institute methodological guidance and reported in accordance with the PRISMA-ScR (Preferred Reporting Items for Systematic Reviews and Meta-Analyses Extension for Scoping Reviews) checklist. A comprehensive search strategy, developed in collaboration with an information specialist, was applied to MEDLINE, Embase, CINAHL, Web of Science, and Google Scholar, without date restrictions. Empirical studies involving adults aged 65 years and older living with neurocognitive disorders and using digital health technologies were included. Study selection and data extraction were performed independently by at least 2 reviewers, and the results were synthesized narratively.

**Results:**

Of the 1665 records identified after duplicate removal, 2 studies met the inclusion criteria. One study examined computerized cognitive stimulation and cognitive engagement programs among community-dwelling older adults with mild neurocognitive disorders, whereas the other explored the use of a personalized digital reminiscence application in long-term care settings among individuals with major neurocognitive disorders. No study used a validated instrument to directly measure engagement. However, observable indicators and markers related to the behavioral, cognitive, and affective components of engagement were reported. Both studies also documented concurrent cognitive or psychosocial outcomes. Factors facilitating engagement included professional support, content personalization, and involvement of informal caregivers, whereas limiting factors included cognitive fluctuations, fatigue, technical complexity, and reliance on external support.

**Conclusions:**

This scoping review highlights a significant gap in the literature regarding the explicit conceptualization and standardized measurement of engagement with digital health technologies among older adults living with neurocognitive disorders. The findings underscore the need to develop and apply multidimensional, context-sensitive engagement measurement tools tailored to this population to better understand and optimize digital health interventions.

## Introduction

### Background

Population aging is a rapidly accelerating global phenomenon [1]. Between 2015 and 2050, the proportion of older adults worldwide is expected to nearly double, increasing from 12% to 22% [1,2]. Aging, defined as the progressive decline of physiological functions necessary for survival and reproduction [3], is frequently associated with an increased prevalence of neurocognitive disorders [4].

Neurocognitive disorders refer to an alteration in one or more cognitive functions, regardless of their underlying mechanism, etiology, or reversibility [5]. Such impairments may result from neurological, psychiatric, pharmacological, or other causes [5] and can affect various cognitive domains, including attention, memory, executive function, visuospatial abilities, and language [5]. In clinical and diagnostic literature, neurocognitive disorders are generally classified into 2 main categories according to the *Diagnostic and Statistical Manual of Mental Disorders* (*DSM-5*) [6]: mild neurocognitive disorder, which corresponds to the concept historically referred to as mild cognitive impairment (MCI), and major neurocognitive disorder, which largely corresponds to what is described as dementia in the International Classification of Diseases, still widely used in the international literature [6,7].

Major neurocognitive disorder is characterized by a significant, acquired, and progressive decline in cognitive abilities that are severe enough to interfere with independence in activities of daily living [5]. In contrast, MCI involves objectively measurable cognitive decline with relatively preserved functional autonomy [5,8].

Although most individuals living with neurocognitive disorders are aged 65 years and older, cognitive disorders can also affect younger populations [9]. In this scoping review, the term “older adults” refers to individuals aged 65 years and older, in accordance with commonly used criteria by statistical and public health organizations in Quebec and Canada [10-12].

Globally, the prevalence of MCI among older adults is estimated to range from 15.6% to 23.7%, depending on community or institutional settings [13]. In addition, approximately 55 million people worldwide are currently living with major neurocognitive disorders, nearly 73% of whom are older adults [14]. This number continues to rise rapidly, with nearly 10 million new cases diagnosed each year [14]. Major neurocognitive disorder encompasses several clinical entities, including Alzheimer disease, which accounts for 60% to 70% of cases, and cerebrovascular diseases, and less common forms, such as frontotemporal degeneration, dementia with Lewy bodies, and mixed neurocognitive disorders [9,14].

In Canada, in 2025, it is estimated that nearly 771,939 individuals may live with major neurocognitive disorders, a number projected to reach approximately 1 million by 2030 and 1.7 million by 2050 [15]. The consequences of neurocognitive disorders, whether mild or major, are substantial for older adults themselves as well as for their informal caregivers (family members and friends) and formal caregivers (health and social care professionals). For example, informal caregivers in Canada provide more than 580 million hours of care annually to older adults living with neurocognitive disorders, equivalent to approximately 290,000 full-time jobs [15]. The associated costs of care and support exceed CAD $10 billion per year [9].

In response to this growing burden, strategies aimed at preserving cognitive health in older adults have become a public health priority. Two key mechanisms have been identified in the literature: cognitive reserve and vascular reserve [16]. Cognitive reserve refers to the brain’s capacity to recruit alternative neural networks and strengthen synaptic connections to compensate for neuropathological damage, thereby maintaining cognitive performance despite underlying brain changes [16,17]. Vascular reserve, in turn, reflects the ability of the cerebral vascular system to maintain adequate oxygen and nutrient delivery despite vascular insults or hemodynamic variations, contributing to overall brain resilience [16,17]. Together, these complementary mechanisms may delay the clinical expression of neurocognitive disorders.

Several protective factors that may influence these mechanisms have been identified, including physical activity, social participation, and cognitive engagement [16,18-20]. These factors can be supported through various interventions, including the use of digital health technologies.

Digital health technologies encompass stand-alone software applications as well as integrated systems and devices accessible via computers, smartphones, tablets, wearable devices, or environmental sensors [21,22]. Among older adults, these technologies, often referred to as gerontechnologies, are used to support multiple aspects of health care and health promotion, such as cognitive stimulation, physical activity monitoring, and communication with family members and health care professionals [4,23].

However, for digital health technologies to achieve their intended benefits, user engagement remains a central determinant. Engagement with digital health technologies is commonly conceptualized as a multidimensional construct comprising 3 interrelated components: behavioral, cognitive, and affective [24-27]. The behavioral component refers to patterns of technology use, including frequency, duration, intensity, and depth of use [26]. The cognitive component encompasses constructs, such as attention and interest directed toward the technology [24,25]. Attention reflects the allocation of cognitive resources to specific information at the expense of competing stimuli [25], whereas interest represents a more enduring preference for certain activities, promoting voluntary engagement and the use of specific cognitive strategies [27]. Finally, the affective component relates to emotional responses elicited by the technology, including hedonic valence (pleasure or displeasure) and emotional activation [27].

Within digital health interventions, technologies function as stimuli designed to reinforce use, attention, interest, and affective responses to support behavior change [28]. Nevertheless, the literature reveals considerable heterogeneity in how engagement with digital health technologies is defined and measured. Engagement is frequently conflated or used interchangeably with related constructs, such as use, adherence, acceptability, satisfaction, feasibility, adoption, or retention [27,29]. In this scoping review, engagement is examined according to the 3-component conceptualization (behavioral, cognitive, and affective) to ensure theoretical consistency [24-27].

Although the effectiveness of digital health technologies for individuals living with neurocognitive disorders has been widely documented [20], knowledge regarding engagement with these technologies among older adults with neurocognitive disorders remains fragmented. Yet, the effectiveness of digital interventions largely depends on their adoption, sustained use, and integration into daily life, all of which require sufficient user engagement [30]. Engagement is therefore considered a key factor in explaining why certain digital health technologies are beneficial for some older adults but not for others [31].

In practice, some older adults may have access to digital health technologies and possess the necessary skills to use them, yet choose not to engage, often due to issues related to design, content, or functionality [26,32]. Others may initially engage but discontinue use over time. For example, a systematic review examining attrition and adherence in mobile mental health interventions among older adults reported attrition rates of up to 30%, with particularly low adherence rates ranging from 2% to 10% [33]. Factors associated with disengagement included dissatisfaction with perceived progress, time and effort demands, and limited usability [30].

Given the limited and heterogeneous evidence on engagement with digital health technologies among older adults living with neurocognitive disorders, there is a clear need to map existing knowledge. A comprehensive understanding of how engagement is conceptualized, assessed, and related to intervention effectiveness is essential to support the development, adaptation, and implementation of more relevant and inclusive digital health interventions for the prevention and management of neurocognitive disorders in older adults.

### Study Objectives and Research Questions

The objective of this scoping review was to describe how engagement with digital health technologies among older adults living with neurocognitive disorders is conceptualized and assessed in the scientific literature. It also aimed to explore factors that facilitate or hinder engagement and to examine the relationship between engagement and the effectiveness of digital health interventions.

The review addressed the following research questions:

1. How is engagement conceptualized and operationalized in studies examining digital health technologies for older adults living with neurocognitive disorders?
2. What measures are used to assess the level of engagement of older adults living with neurocognitive disorders in digital health technologies?
3. What factors facilitate or hinder engagement with digital health technologies among older adults living with neurocognitive disorders?
4. What relationships are reported between engagement levels and the effectiveness of digital health interventions?

## Methods

### Study Design

This study adopted a scoping review methodology to map the existing body of knowledge, clarify conceptual boundaries, and identify gaps in the literature regarding engagement with digital health technologies among older adults living with neurocognitive disorders [34]. Scoping reviews are particularly appropriate when concepts are heterogeneous, variably defined, and examined using diverse methodological approaches [34].

We conducted the review following the Joanna Briggs Institute (JBI) methodological guidance for scoping reviews [35]. Reporting followed the PRISMA-ScR (Preferred Reporting Items for Systematic Reviews and Meta-Analyses Extension for Scoping Reviews) checklist (Checklist 1) [36] to ensure transparency and reproducibility.

### Eligibility Criteria

Eligibility criteria were defined a priori using the Participants (Population), Concept, and Context framework recommended by the JBI [35].

#### Population

Studies involving older adults aged 65 years and older living with neurocognitive disorders, whether mild or major, were considered eligible. Given the variability in how age was reported across studies, exceptions were allowed. Specifically, studies were included when the mean or median age of participants was greater than 65 years [37]. Furthermore, several conceptual frameworks and recent evidence syntheses indicate that the involvement of caregivers, whether formal or informal, is a key determinant of digital engagement among individuals living with a neurocognitive disorder. This is largely due to their role in content personalization, support for technology use, and technological mediation between the user and the digital tool [38-40]. Accordingly, although caregivers were not considered the primary population of interest in this review, their data were included when they directly informed the engagement of older adults living with a neurocognitive disorder in the use of digital health technologies.

#### Concept

The central concept of this review was engagement with digital health technologies. No restrictions were imposed on how engagement was defined in the included studies, in order to capture the diversity of conceptualizations present in the literature. Digital health technologies included stand-alone software applications as well as integrated systems and devices accessible via platforms, such as computers, smartphones, tablets, wearable devices, and environmental sensors [21,22].

#### Context

Studies conducted in any setting (eg, health care facilities, long-term care settings, residential facilities for older adults, or community-based environments) were eligible for inclusion, with no geographical restrictions.

### Exclusion Criteria

Publications that did not report original empirical data were excluded. These included knowledge syntheses, such as systematic reviews and narrative reviews, as well as commentaries, editorials, and study protocols. Conference abstracts, theses, dissertations, and gray literature were also excluded.

### Search Strategy

The search strategy (Multimedia Appendix 1) was developed in collaboration with an experienced health sciences librarian specialized in knowledge synthesis (FB). It combined free-text terms and controlled vocabulary related to the concepts of *engagement*, *digital health*, *older adults*, and *neurocognitive disorders*, including relevant synonyms and spelling variants.

An initial comprehensive search was conducted on April 4, 2024, and updated on October 9, 2025. The databases searched included MEDLINE (Ovid), Embase, CINAHL, Web of Science, and Google Scholar. Considering the known limitations of search engines and based on preliminary testing, we elected to perform a series of structured subsearches [41]. To maximize the effectiveness of the relevance-ranking algorithm, each subsearch was restricted to the top 20 results. In total, this strategy produced 160 records, including those retrieved during the update. Only studies published in English or French were included. No other restrictions were applied. Finally, backward citation searching was performed on all included studies to identify further relevant publications.

### Study Selection

All records retrieved from the databases were imported into Covidence (Veritas Health Innovation), a web-based collaborative software designed to manage systematic and scoping reviews [42]. A total of 780 duplicates were eliminated using the automated function of Covidence, and 5 duplicates were removed manually.

Study selection was conducted in two stages: (1) title and abstract screening and (2) full-text review. At each stage, eligibility criteria were applied independently and blindly by several reviewers (SMARD, FN, SA, ARY, FY, JP, and CC). Discrepancies were resolved through discussion and consensus, and when necessary, by consultation with the senior investigator (MPG).

A PRISMA 2020 flow diagram was used to document the identification, screening, eligibility assessment, and inclusion of studies, as well as reasons for exclusion at the full-text review stage [43].

### Data Extraction

A standardized data extraction form was developed by the research team using Microsoft Excel. Data extraction was performed independently by several reviewers (SMARD, ARY, FN, and SA) and subsequently validated by the senior investigator (MPG). Discrepancies were resolved through consensus among the reviewers involved or, when needed, with the support of a third team member.

The following data were extracted: (1) study and intervention characteristics, such as title, authors, year of publication, country, study design, eligibility criteria, care setting, target population, type of digital health technology, duration, and reported effectiveness; and (2) engagement-related variables, such as conceptualization of engagement, operationalization of engagement, engagement indicators (behavioral, cognitive, and affective), and measurement tools used.

In addition, variables derived from the PROGRESS-Plus framework for health equity were extracted [32]. This framework includes 8 core dimensions (place of residence, race/ethnicity, occupation, gender, religion, education, socioeconomic status, and social capital) and an additional “Plus” component intended to capture other characteristics that may influence health inequities. The use of this framework allowed for an examination of the extent to which digital health technologies were accessible and adapted to potentially vulnerable subgroups, including older adults living with neurocognitive disorders [44].

### Data Analysis

A descriptive analysis was conducted to synthesize the characteristics of the included studies, which were presented in a tabular form.

To synthesize findings on engagement with digital health technologies, we used a 3-dimensional conceptualization of engagement (behavioral, cognitive, and affective) as a descriptive analytical tool [24-27]. This framework served solely to organize and categorize elements reported in the included studies, without assessing the relevance, validity, or psychometric properties of the conceptual model. This approach aligns fully with the purpose of a scoping review, which aims to map concepts, definitions, and operationalization modes of engagement in the existing literature [34].

Variables reported in the included studies were recoded a posteriori by the research team, under the supervision of the principal investigator (MPG), into engagement indicators corresponding to the 3 components, to ensure a theoretically coherent interpretation of findings derived from heterogeneous study designs.

We did not assess the methodological quality of the included studies, in accordance with the JBI guidance for scoping reviews [35]. The objective of this review was to map existing evidence rather than evaluate effectiveness or draw conclusions based on the level of evidence.

## Results

### Study Selection

**Figure 1.**
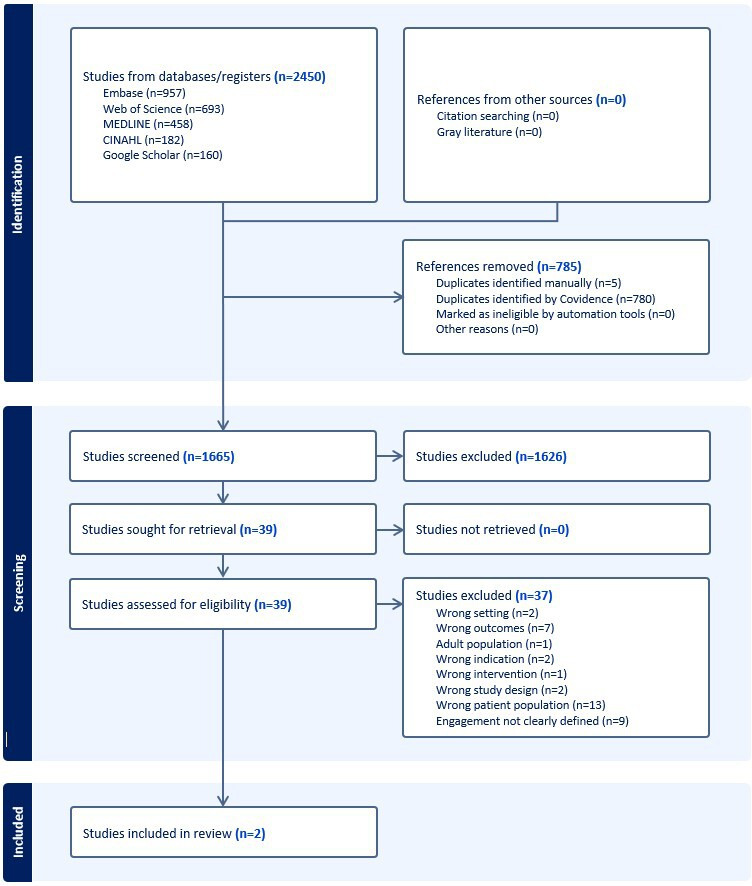
PRISMA (Preferred Reporting Items for Systematic Reviews and Meta-Analyses) 2020 flowchart for study inclusion [43].

The search strategy identified a total of 1665 records after the removal of duplicates (Figure 1). During the first screening stage, based on titles and abstracts, 37 publications were retained for full-text review. Following full-text assessment, 2 publications [45,46], reporting on 2 distinct studies, met all eligibility criteria and were included in the scoping review.

### Study Characteristics

The first study [45] assessed the feasibility and acceptability of 2 distinct digital interventions among older adults living with MCI. It compared a computerized cognitive stimulation (CCS) program with a computerized cognitive engagement (CCE) program. The CCS program aimed to stimulate multiple cognitive domains through computerized cognitive exercises combined with structured social interactions among participants. In contrast, the CCE program focused on learning to use a digital tablet, combined with the stimulation of social interactions within the group.

This study used a single-blind randomized controlled trial design. Participants were community-dwelling adults aged 60 years and older with MCI, recruited from a memory clinic in France. A total of 20 participants were randomly and equally allocated to the CCS and CCE groups. The mean age was 78.2 (SD 7.0) years in the CCE group and 75.2 (SD 6.4) years in the CCS group. Both groups participated in weekly group sessions lasting 90 minutes over a 3-month period. Sessions were facilitated by a trained neuropsychologist who was blinded to the outcome assessments.

The second study [46] explored strategies to enhance engagement among older adults living with dementia in a long-term care setting using a digital reminiscence application called *Memory Keeper*. This application, developed by the study authors, aimed to support well-being and engagement among individuals with dementia by stimulating personal memories through personalized multimedia content.

The authors examined barriers and facilitators to using the application, perceived benefits of its integration into long-term care, and its potential use within existing care practices. Participants included 3 residents (2 men and 1 woman) aged 76 to 87 years with major neurocognitive disorders, 6 informal caregivers (family members or close relatives), and 1 formal caregiver (the facility’s lifestyle coordinator). Participants were recruited using purposive sampling.

Informal caregivers contributed to the creation of personalized content (photographs and image libraries) integrated into the application and were trained in its use. Among the residents with major neurocognitive disorders, 1 had a diagnosis of dementia with Lewy bodies, 1 had Alzheimer disease, and 1 had dementia of an unspecified type. Although the study design was not explicitly stated in the *Methods* section, data collection and analysis procedures were qualitative in nature (see Table 1 for detailed study characteristics).

Table 1 presents the main characteristics of the included studies, including country and year of publication, study population, type of neurocognitive disorders, digital health intervention, study design, duration, and setting.

**Table 1.** Characteristics of included studies.

Study and year	Country	Population	Type of neurocognitive disorders	Intervention	Design	Duration	Setting
Djabelkhir et al [45] (2017)	France	20 community-dwelling older adults; mean age >75 years; CCE^a^: 78.2 (SD 7) years; CCS:^b^ 75.2 (SD 6.4) years	MCI^c^	CCS versus CCE	Randomized controlled trial, single-blind	3 months, weekly 90-min sessions	Community
McAllister et al [46] (2020)	Australia	3 residents (ages 76‐87 y); 6 informal caregivers; n=1 formal caregiver	Dementia: Alzheimer, Lewy body, and unspecified	Digital reminiscence application (Memory Keeper)	Qualitative methods	Duration not specified (real-world exploration)	Long-term care facility

a CCE: computerized cognitive engagement.

b CCS: computerized cognitive stimulation.

c MCI: mild cognitive impairment.

### Conceptualization and Operationalization of Engagement With Digital Health Technologies

#### Conceptualization of Engagement

Both included studies [45,46] conceptualized engagement as a sustained interaction between older adults living with neurocognitive disorders and digital health technologies, mediated by digital stimuli and embedded within a social dimension.

More specifically, the study involving older adults with MCI [45] conceptualized engagement through participation in cognitively stimulating group-based activities combining computerized cognitive exercises and structured social interactions. In contrast, the study conducted among individuals with dementia in long-term care [46] conceptualized engagement as observable reactions and dyadic interactions elicited by personalized digital stimuli (reminiscence content) within a caregiving context.

#### Operationalization of Engagement

Engagement with digital health technologies was operationalized heterogeneously across the included studies [45,46]. However, the analytical recoding conducted in this scoping review enabled the alignment of all reported indicators with the 3-component conceptualization of engagement (behavioral, cognitive, and affective) [24-27], which was used as the analytical framework.

It is important to note that this 3-component analytical framework was applied a posteriori to heterogeneous data for analytical purposes rather than evaluative assessment, in accordance with the objectives of a scoping review.

At the behavioral level, engagement was documented through indicators, such as session attendance and attrition [45]. In the long-term care context, behavioral engagement was reflected by the frequency and duration of visits and by observable interaction-related behaviors during application use, including dancing or tapping movements [46]. The cognitive component of engagement was operationalized using standardized neuropsychological tests, including the Trail Making Test Part A (TMT-A, attentional speed), Trail Making Test Part B (TMT-B, error reduction), free recall tasks (RL/RI-16), and the visuospatial memory test [45]. In another study, cognitive engagement was reflected by qualitative markers, such as sustained attention to the application and spontaneous recall of names or details associated with personalized reminiscence content [46]. Finally, the affective component of engagement was operationalized through indicators, such as motivation, self-esteem, and technology acceptance [45]. In the long-term care context, affective engagement was reflected by observable emotional markers, including smiling, singing, positive facial expressions, absence of negative reactions, and visits perceived as more pleasant by caregivers [46].

### Measurement of Engagement

This section describes the approaches used to measure engagement as reported in the included studies and highlights the diversity of methods used to discuss capture engagement with digital health technologies among older adults living with neurocognitive disorders. It also specifies the participant groups to which the different measures were applied, in order to reflect study-specific data collection modalities, as well as the engagement components documented in the existing literature.

None of the included studies [45,46] used a validated instrument specifically designed to directly measure engagement with digital health technologies. However, in a study by Djabelkhir et al [45], a combination of validated instruments was administered to participants in the CCE and CCS groups to assess selected indicators associated with engagement. Executive functions were measured using the TMT-A and TMT-B. Episodic memory was assessed using the RL/RI-16 free and cued recall test as well as the visuospatial memory test from the cognitive efficiency profile. Global self-esteem was measured using the Rosenberg Self-Esteem Scale, and acceptance of information and communication technologies was assessed using a technology acceptance questionnaire. In the other study [46], engagement among residents living with dementia was documented using observable qualitative markers, primarily collected during interactions with the application, based on observations reported by caregivers and staff.

From an analytical perspective, the reported measures were subsequently examined according to the behavioral, cognitive, and affective components of engagement, while considering the participant groups associated with each type of measure.

The behavioral component was assessed using participation- and use-related measures collected from participants in the CCE and CCS groups in the study by Djabelkhir et al [45], and from residents in the study by McAllister et al [46], including indicators related to attendance, attrition, and the frequency and duration of interactions, supplemented by observations of interaction-related behaviors.

The cognitive component was documented through cognitive performance assessments administered to participants in the CCE and CCS groups, as well as through qualitative markers reflecting attention and memory processes mobilized by residents during technology use.

Finally, the affective component was captured using psychosocial indicators collected from participants in the CCE and CCS groups and through observational markers documenting emotional responses and residents’ perceived experiences during interactions, as reported by caregivers.

Together, these approaches allowed engagement to be examined across behavioral, cognitive, and affective components (Table 2).

**Table 2.** Indicators and markers by engagement component.

Engagement component and type of measure	Participant groups	Instruments/markers used
Behavioral		
Participation measures	Participants in the CCE^a^ and CCS^b^ groups (study [45])	Attendance at activities and attrition
Use-related measures	Residents living with dementia (study [46])	Frequency and duration of interactions with the application
Behavioral observation	Residents, observations reported by caregivers and staff (study [46])	Interaction-related behaviors with the technology (eg, movements and gestures)
Cognitive		
Cognitive performance assessments	Participants in the CCE and CCS groups (study [45])	Trail Making Test (parts A and B), free and cued recall (RL/RI-16), and VST^c^
Qualitative cognitive markers	Residents, observations by caregivers and staff (study [46])	Attention to the application and engagement of memory processes during interactions
Affective		
Psychosocial indicators	Participants in the CCE and CCS groups (study [45])	Motivation, self-esteem (Rosenberg Self-Esteem Scale), and technology acceptance
Observational affective markers	Residents, observations reported by caregivers (study [46])	Emotional responses and perceived experience during interactions

a CCE*:* computerized cognitive engagement.

b CCS*:* computerized cognitive stimulation.

c VST: visuospatial memory test.

### Relationship Between Engagement and the Effectiveness of Digital Health Interventions

Across the included studies, different analytical approaches were used to present the findings. A study by Djabelkhir et al [45] adopted a quantitative approach, relying on pre-post statistical analyses to examine cognitive and psychosocial data collected from participants in the CCE and CCS groups; for selected indicators, effect sizes were also reported to characterize the magnitude of the observed changes. In contrast, a study by McAllister et al [46] used a qualitative descriptive approach and used a framework analysis to structure and synthesize observational data derived from interactions between residents living with dementia and the digital application, *Memory Keeper*.

In this scoping review, these analytical methods are reported for descriptive purposes only, to contextualize how the findings were generated, without inferring any causal relationship between engagement and the reported outcomes.

Both included studies [45,46] provided data that enabled the concurrent examination of engagement manifestations and cognitive or psychosocial outcomes associated with digital health interventions among older adults living with neurocognitive disorders, across different contexts and levels of severity.

In the study involving older adults with MCI [45], sustained engagement with digital health interventions was observed alongside improvements in cognitive performance. These improvements included gains on the TMT-A and TMT-B, a medium effect size for free recall, and a trend toward improvement in visuospatial memory.

In the long-term care study involving older adults with dementia [46], engagement was not assessed using standardized measures. However, qualitative observations indicated that active participation by residents, characterized by sustained attention, recall of personal details, and increased interactions with caregivers, coincided with positive experiences during the use of the digital reminiscence application.

At the affective level, both studies reported convergent indicators [45,46]. High motivation, improved self-esteem, and increased technology acceptance were documented in a study by Djabelkhir et al [45], whereas the other study reported positive emotional expressions, interactions perceived as more pleasant, and an absence of negative reactions during the application use [46].

### Facilitating and Limiting Factors of Engagement With Digital Health Technologies

#### Overview

The facilitating and limiting factors of engagement with digital health technologies identified in the included studies are presented in Table 3. These factors were identified transversally based on the data reported in the studies [45,46] and interpreted in light of the 3-component conceptualization of engagement (behavioral, cognitive, and affective), which was used as the analytical framework in this scoping review.

**Table 3.** Facilitators and barriers to engagement.

Factor type and reported factors	Engagement components involved	Studies
Facilitating		
Structured and professionally supervised activities, integration of technologies into existing contexts (group sessions and family visits), and intuitive interfaces	Behavioral	[45,46]
Guided learning, task adaptation, and personalization of digital content	Cognitive	[45,46]
Motivation, sense of personal value, and positive technology acceptance	Affective	[45]
Limiting		
Time constraints, reliance on caregiver or staff availability, and perceived activity duration	Behavioral	[45,46]
Fatigue, drowsiness, and fluctuations in cognitive state and attention	Cognitive	[46]
Loss of personal relevance of content, sedated states, and emotional disengagement	Affective	[46]

#### Facilitating Factors of Engagement

Several facilitating factors of engagement were reported across the included studies, although their nature varied according to the intervention context and the population studied.

At the behavioral level, engagement was facilitated by participation in structured and professionally supervised activities as well as by the integration of technologies into existing use contexts, such as group sessions in community settings [45] or family visits and interactions with staff in long-term care settings [46]. Ease of use and the intuitive nature of digital interfaces were also reported as factors facilitating interaction with technologies [45,46].

At the cognitive level, facilitating factors included guided learning in the use of technologies and the adaptation of tasks or digital content to participants’ abilities [45]. The personalization of digital content, particularly in the form of reminiscence materials, was also described as supporting attention and cognitive engagement, especially among individuals living with dementia [46].

Finally, at the affective level, facilitating factors, such as sustained motivation to participate, a sense of personal value, and positive acceptance of digital technologies, were reported [45]. These elements were primarily described in the study conducted among older adults living with MCI.

#### Limiting Factors of Engagement

The included studies also identified several limiting factors of engagement with digital health technologies. At the behavioral level, organizational constraints, such as limited time available for technology use or reliance on the availability of caregivers or staff to initiate use, were reported as barriers to engagement [46]. In some cases, the perceived duration of certain activities was also identified as a source of frustration [45].

At the cognitive level, fluctuations in the cognitive state, including fatigue, drowsiness, or variations in attention, were described as factors limiting engagement, particularly among individuals living with dementia [46]. These factors could restrict the ability to maintain sustained interaction with the technologies.

Finally, at the affective level, limiting factors were reported when digital content lost its personal relevance or meaning for participants [46]. Sedated states or transient emotional disengagement were also mentioned as elements likely to limit the experience perceived as positive during technology use [46].

Facilitating and limiting factors are presented in Table 3.

## Discussion

This scoping review aimed to examine how engagement with digital health technologies is conceptualized, operationalized, and measured among older adults living with mild or major neurocognitive disorders and to explore its relationship with intervention effectiveness.

### Principal Findings

The findings highlight a major gap in the scientific literature regarding engagement with digital health technologies among older adults living with neurocognitive disorders. Despite a comprehensive search strategy across multiple databases, only 2 empirical studies [45,46] met the inclusion criteria among the 1665 records identified after the removal of duplicates. These findings suggest that empirical research explicitly addressing engagement in older adults living with neurocognitive disorders remains limited. The results show that although engagement was neither explicitly defined nor directly measured in the included studies, the reported data encompassed all dimensions commonly associated with engagement in digital health, as described in the literature. These manifestations included high attendance and low attrition, signs of sustained attention and cognitive mobilization, and positive affective responses during technology use. In addition, this review identified several factors that facilitated or hindered engagement, which were related to technological characteristics, individual factors, and contextual conditions.

Although the studies reported engagement-related indicators alongside cognitive or psychosocial outcomes, the absence of standardized and explicit engagement measures precludes any causal interpretation. These findings highlight the need to better conceptualize, operationalize, and measure engagement with digital health technologies among older adults living with neurocognitive disorders, while accounting for individual, social, and contextual determinants.

### Conceptualization and Operationalization of Engagement

The findings of this review indicate that engagement with digital health technologies among older adults living with neurocognitive disorders remains variably and often implicitly conceptualized. This observation is consistent with previous literature showing that engagement is frequently conflated with related constructs, such as use, adherence, acceptability, or retention, rather than being treated as a distinct concept [29,31].

In the included studies, engagement was implicitly conceptualized as sustained interaction between the individual and digital stimuli, embedded within a social context (group-based or dyadic). This implicit conceptualization aligns with dominant theoretical frameworks in digital health, which describe engagement as a multidimensional process comprising behavioral, cognitive, and affective components [22,24-27].

Although the 3-component conceptualization of engagement was not explicitly adopted by the included studies, the results indicate that the observed operationalization, reconstructed a posteriori in this review, covered all 3 components. This suggests that among older adults living with neurocognitive disorders, engagement extends beyond mere technical use and encompasses central cognitive and affective dimensions.

Recent methodological work emphasizes the importance of adapting engagement measurement to the context of use and study objectives, acknowledging that available tools variably capture different engagement components [25,26]. A key methodological contribution of this review lies in the application of a 3-component analytical framework to harmonize findings derived from heterogeneous study designs. This approach highlights that, in older adults living with mild or major neurocognitive disorders, observable affective responses (eg, smiling and singing) may be as informative as traditional behavioral usage metrics [24,26,28].

### Measurement of Engagement

None of the included studies used a validated instrument specifically designed to measure engagement with digital health technologies in older adults living with neurocognitive disorders. This limitation mirrors broader challenges in the digital health literature, including the lack of methodological consensus and the predominance of indirect indicators [22,27].

In long-term care contexts, the use of validated observational tools, such as those developed to assess engagement among individuals with neurocognitive disorders [28], could allow for a more nuanced assessment of experiential engagement, particularly among individuals with major neurocognitive disorders. The absence of standardized engagement measures limits comparability across studies and constrains the ability to formally examine engagement as a mechanism of action in digital interventions.

### Facilitating and Limiting Factors: The Central Role of Context and Human Support

Facilitating factors identified in this review included content personalization, ease of use, professional supervision, and involvement of informal caregivers. These findings are consistent with user-centered models emphasizing alignment between individual capacities, technological design, and contextual conditions [24,47].

Conversely, several limiting factors were identified, including technical complexity, loss of personal relevance of content, fatigue, drowsiness, fluctuations in cognitive state, and organizational constraints in care settings. Reliance on external support to initiate technology use emerged as a particularly important barrier, underscoring that engagement cannot be considered independently of the social and institutional environments in which technologies are deployed.

### Engagement and Effectiveness of Digital Health Interventions

Although the number of included studies was limited, the available data allow for a cautious discussion of the potential link between engagement and intervention effectiveness. The studies reported concurrent engagement manifestations and cognitive or psychosocial outcomes, without enabling causal or directional conclusions. This limitation is consistent with the broader digital health literature, in which engagement is often assumed to be a key mechanism but is rarely empirically tested as such [22,30].

These observations align with a wider body of evidence suggesting that well-designed and appropriately adapted digital interventions may yield modest to moderate cognitive benefits among individuals living with neurocognitive disorders, despite substantial methodological heterogeneity [48,49]. However, the included studies were not designed to formally test engagement as a mediator or moderator, and any association between engagement and effectiveness must therefore be interpreted cautiously.

### Strengths and Limitations

This scoping review has several strengths. First, a major strength lies in its theoretically informed analytical approach. Engagement-related data were recoded a posteriori using a 3-component conceptualization of engagement (behavioral, cognitive, and affective), which served as an analytical framework to harmonize heterogeneous data without aiming to validate or test this conceptual framework. This approach is particularly relevant in a research field characterized by substantial conceptual and methodological variability. Second, the review explicitly incorporated a health equity perspective using the PROGRESS-Plus framework. Although such information was rarely reported in the included studies, its systematic consideration allowed for the identification of an important gap in the literature and strengthened the scope of the evidence mapping.

Despite these strengths, several limitations should be acknowledged. First, the available evidence is constrained by small sample sizes, the underrepresentation of individuals living with major neurocognitive disorders, heterogeneous study designs, and the absence of standardized instruments specifically developed to measure engagement with digital health technologies. These factors limit the robustness and comparability of the findings. Moreover, engagement was most often inferred or reconstructed a posteriori rather than explicitly defined and systematically measured as a primary construct. Second, considerations related to health equity were largely absent from the included studies. Variables encompassed within the PROGRESS-Plus framework were infrequently reported, limiting the examination of how social, demographic, and contextual factors may influence engagement and restricting the generalizability of the findings to diverse populations of older adults. Third, the limited number of eligible studies and their predominantly exploratory nature reflect the early stage of development of this field of research. Although this limits the scope of conclusions that can be drawn, it also underscores the appropriateness of adopting a scoping review approach to map existing evidence, identify gaps, and inform future research directions.

### Future Directions

The findings of this review have implications for both practice and research. The identified facilitating factors, particularly content personalization, professional supervision, and caregiver involvement, suggest that digital health technologies for older adults living with mild or major neurocognitive disorders should be developed and implemented in close alignment with users’ cognitive capacities, care contexts, and available support networks.

The findings also highlight the need to develop validated, multidimensional engagement measurement tools tailored to specific contexts of use and to conduct larger-scale studies that integrate equity considerations.

### Conclusion

This scoping review highlights a substantial gap between the theoretical recognition of engagement as a key determinant of the effectiveness of digital health technologies and its empirical operationalization among older adults living with neurocognitive disorders. It shows that engagement with digital health technologies in this population remains an emerging research area, characterized by significant methodological challenges and considerable potential for development.

There is a critical need to develop contextualized, rigorous, and population-sensitive engagement measurement tools as well as to ensure the systematic and explicit integration of the behavioral, cognitive, and affective dimensions of engagement. Addressing these priorities is essential to strengthen the design, evaluation, and implementation of digital health interventions that are more relevant and better tailored to supporting cognitive health among older adults living with neurocognitive disorders.

## Supplementary material

10.2196/70157 Multimedia Appendix 1 Search strategy.

10.2196/70157 Multimedia Appendix 2 Research strategy.

10.2196/70157 Checklist 1 PRISMA-ScR checklist.

## Abbreviations

CCE: computerized cognitive engagement
CCS: computerized cognitive stimulation
DSM-5: Diagnostic and Statistical Manual of Mental Disorders, Fifth Edition
JBI: Joanna Briggs Institute
MCI: mild cognitive impairment
PRISMA-ScR: Preferred Reporting Items for Systematic Reviews and Meta-Analyses Extension for Scoping Reviews
TMT: Trail Making Test
