# Supplementary material for: Engaging Older Adults With Neurocognitive Disorders in Digital Health Technologies: Scoping Review
Source: JMIR Hum Factors. 2026 Jul 17;13:e70157. doi: 10.2196/70157 (PMC13378898; doi:10.2196/70157)
Supplement: Multimedia Appendix 1 [file humanfactors-v13-e70157-s001.docx]

**Supplementary materials – Databases search strategy**

**Medline (Ovid)**

**Date of the initial search:** 23-04-2024

**Date of the updated search**: 09-10-2025

**Database limit:** no database limit has been applied.

| **#** | **Search strategy** | **Results** |
| --- | --- | --- |
| 1 | Patient Participation/ OR (Engage* OR Involv* OR Participation OR disengag*).ti,ab,kf OR (microengagement OR macroengagement).ti,ab,kf |  |
| 2 | Digital Technology/ OR Digital Health/ OR Smartphone/ OR Computers, Handheld/ OR Mobile Applications/ OR (Digital adj1 (Technolog* OR Health)).ti,ab,kf OR (mobile adj2 (application? OR technolog* OR app OR device? OR apps OR Phone)).ti,ab,kf OR Smartphone?.ti,ab,kf  OR (tablet adj1 (application? OR computer?)).ti,ab,kf OR "chat bot?".ti,ab,kf  OR chatterbot?.ti,ab,kf OR chatbot?.ti,ab,kf OR medbot?.ti,ab,kf OR "chatter bot?".ti,ab,kf OR smart bot?.ti,ab,kf OR smartbot?.ti,ab,kf OR (Conversational adj2 (assistant OR interface OR agent? OR system OR computer OR bot? OR AI)).ti,ab,kf OR ((virtual OR intelligent OR computer OR AI OR "artificial intelligence" OR embodied) adj2 agent?).ti,ab,kf |  |
| 3 | exp Aged/ OR Aged.ti,ab,kf OR geriatric?.ti,ab,kf OR elder*.ti,ab,kf OR senior?.ti,ab,kf OR ((old OR oldest? OR older?) adj2 (person? OR adult? OR individual? OR people OR patient?)).ti,ab,kf |  |
| 4 | Mental Disorders/ OR exp Neurocognitive Disorders/ OR Memory Disorders/ OR Schizophrenia/ OR exp Parkinsonian Disorders/  OR ((Mental OR Neurocogniti* OR Psych* OR Cogniti*) adj2 (Disorder? OR Illness* OR Disease? OR Dysfunction OR Decline OR Deterioration OR Impairment?)).ti,ab,kf OR (Memory adj2 (Disorder? OR Deficit OR Loss* OR Impairment?)).ti,ab,kf OR Alzheimer?.ti,ab,kf OR Schizophrenia?.ti,ab,kf  OR Parkinson?.ti,ab,kf OR Huntington?.ti,ab,kf OR Delirium.ti,ab,kf OR dementia?.ti,ab,kf OR Amnesia?.ti,ab,kf OR (Lewy adj1 (Body OR Bodies)).ti,ab,kf |  |
| 5 | 1 AND 2 AND 3 AND 4 | 278 |
| 6 | limit 5 to ez=20240424-20251009 OR limit 5 to dt=20240424-20251009 OR limit 5 to ed=20240424-20251009 | 180 |
|  | **Total number of results** | 458 |

**Embase (Embase.com)**

**Date of the initial search:** 23-04-2024

**Date of the updated search**: 09-10-2025

**Database limit:** no database limit has been applied.

| **#** | **Search strategy** | **Results** |
| --- | --- | --- |
| 1 | 'patient participation'/de OR (Engage* OR Involv* OR Participation OR disengag*):ti,ab,kw OR (microengagement OR macroengagement):ti,ab,kw |  |
| 2 | 'digital technology'/de OR 'digital health'/exp OR 'mobile phone'/exp OR 'personal digital assistant'/de OR 'mobile application'/exp OR 'chatbot'/exp OR (Digital NEAR/1 (Technolog* OR Health)):ti,ab,kw OR (mobile NEAR/2 (application$ OR technolog* OR app OR device$ OR apps OR Phone)):ti,ab,kw OR Smartphone$:ti,ab,kw OR (tablet NEAR/1 (application$ OR computer$)):ti,ab,kw OR "chat bot$":ti,ab,kw OR chatterbot$:ti,ab,kw OR chatbot$:ti,ab,kw OR medbot$:ti,ab,kw OR "chatter bot$":ti,ab,kw OR "smart bot$":ti,ab,kw OR smartbot$:ti,ab,kw OR (Conversational NEAR/2 (assistant OR interface OR agent$ OR system OR computer OR bot$ OR AI)):ti,ab,kw  OR ((virtual OR intelligent OR computer OR AI OR "artificial intelligence" OR embodied) NEAR/2 agent$):ti,ab,kw |  |
| 3 | 'aged'/exp OR Aged:ti,ab,kw OR geriatric$:ti,ab,kw OR elder*:ti,ab,kw OR senior$:ti,ab,kw OR ((old OR oldest$ OR older$) NEAR/2 (person$ OR adult$ OR individual$ OR people OR patient$)):ti,ab,kw |  |
| 4 | 'mental disease'/de OR 'mild cognitive impairment'/de OR 'memory disorder'/exp OR 'delirium'/de OR 'cognitive defect'/de OR 'dementia'/de OR 'Alzheimer disease'/de OR 'Huntington chorea'/de OR 'schizophrenia'/de  OR 'Parkinson disease'/de OR 'parkinsonism'/de OR ((Mental OR Neurocogniti* OR Psych* OR Cogniti*) NEAR/2 (Disorder$ OR Illness* OR Disease$ OR Dysfunction OR Decline OR Deterioration OR Impairment$)):ti,ab,kw OR (Memory NEAR/2 (Disorder$ OR Deficit OR Loss* OR Impairment$)):ti,ab,kw OR Alzheimer$:ti,ab,kw OR Schizophrenia$:ti,ab,kw OR Parkinson$:ti,ab,kw OR Huntington$:ti,ab,kw  OR Delirium:ti,ab,kw OR dementia$:ti,ab,kw OR Amnesia$:ti,ab,kw OR (Lewy NEAR/1 (Body OR Bodies)):ti,ab,kw |  |
| 5 | #1 AND #2 AND #3 AND #4 | 425 |
| 6 | #5 AND [24-04-2024]/sd | 532 |
|  | **Total number of results** | 957 |

**CINAHL (EBSCO)**

**Date of the initial search:** 23-04-2024

**Date of the updated search**: 09-10-2025

**Database limit:** no database limit has been applied.

| **#** | **Search strategy** | **Results** |
| --- | --- | --- |
| 1 | MH "Patient Participation" OR TI (Engage* OR Involv* OR Participation OR disengag*) OR AB (Engage* OR Involv* OR Participation OR disengag*)) |  |
| 2 | MH "Digital Technology" OR MH "Digital Health" OR MH Smartphone  OR MH "Mobile Applications" OR MH "Computers, Hand-Held" OR TI (Digital N1 (Technolog* OR Health)) OR AB (Digital N1 (Technolog* OR Health))  OR TI (mobile N2 (application# OR technolog* OR app OR device# OR apps OR Phone)) OR AB (mobile N2 (application# OR technolog* OR app OR device# OR apps OR Phone)) OR TI Smartphone# OR AB Smartphone# OR TI (tablet N1 (application# OR computer#)) OR AB (tablet N1 (application# OR computer#)) OR TI "chat bot#" OR AB "chat bot#" OR TI chatterbot# OR AB chatterbot# OR TI chatbot# OR AB chatbot# OR TI medbot# OR AB medbot# OR TI "chatter bot#" OR AB "chatter bot#" OR TI "smart bot#"  OR AB "smart bot#" OR TI smartbot# OR AB smartbot# OR TI (Conversational N2 (assistant OR interface OR agent# OR system OR computer OR bot# OR AI)) OR AB (Conversational N2 (assistant OR interface OR agent# OR system OR computer OR bot# OR AI)) OR TI ((virtual OR intelligent OR computer OR AI OR "artificial intelligence" OR embodied) N2 agent#) OR AB ((virtual OR intelligent OR computer OR AI OR "artificial intelligence" OR embodied) N2 agent#) |  |
| 3 | MH "Aged+" OR TI Aged OR AB Aged OR TI geriatric# OR AB geriatric# OR TI elder* OR AB elder* OR TI senior# OR AB senior# OR TI ((old OR oldest? OR older#) N2 (person# OR adult# OR individual# OR people OR patient#))  OR AB ((old OR oldest? OR older#) N2 (person# OR adult# OR individual# OR people OR patient#)) |  |
| 4 | MH "Mental Disorders" OR MH "Mental Disorders, Chronic" OR MH "Alzheimer's Disease" OR MH "Dementia" OR MH "Delirium" OR MH "Mild Cognitive Impairment" OR MH "Cognition Disorders" OR MH "Amnesia" OR MH "Memory Disorders" OR MH Schizophrenia OR MH "Parkinsonian Disorders" OR MH "Lewy Body Disease" OR MH "Parkinson Disease"  OR TI ((Mental OR Neurocogniti* OR Psych* OR Cogniti*) N2 (Disorder# OR Illness* OR Disease# OR Dysfunction OR Decline OR Deterioration OR Impairment#)) OR AB ((Mental OR Neurocogniti* OR Psych* OR Cogniti*) N2 (Disorder# OR Illness* OR Disease# OR Dysfunction OR Decline OR Deterioration OR Impairment#)) OR TI (Memory N2 (Disorder# OR Deficit OR Loss* OR Impairment#)) OR AB (Memory N2 (Disorder# OR Deficit OR Loss* OR Impairment#)) OR TI Alzheimer# OR AB Alzheimer# OR TI Schizophrenia# OR AB Schizophrenia# OR TI Parkinson# OR AB Parkinson# OR TI Huntington# OR AB Huntington# OR TI Delirium OR AB Delirium OR TI dementia# OR AB dementia# OR TI Amnesia# OR AB Amnesia# OR TI (Lewy N1 (Body OR Bodies)) OR AB (Lewy N1 (Body OR Bodies)) |  |
| 5 | S1 AND S2 AND S3 AND S4 | 127 |
| 6 | S5 AND EM 20240424-20251009 | 55 |
|  | **Total number of results** | 182 |

**Web of Science**

**Date of the initial search:** 23-04-2024

**Date of the updated search**: 09-10-2025

**Database limit:** no database limit has been applied.

| **#** | **Search strategy** | **Results** |
| --- | --- | --- |
| 1 | TS=(Engage* OR Involv* OR Participation OR disengag*) OR TS=(microengagement OR macroengagement) |  |
| 2 | TS=(Digital NEAR/1 (Technolog* OR Health)) OR TS=(mobile NEAR/2 (application$ OR technolog* OR app OR device$ OR apps OR Phone)) OR TS=Smartphone$ OR TS=(tablet NEAR/1 (application$ OR computer$)) OR TS="chat bot$" OR TS=chatterbot$ OR TS=chatbot$ OR TS=medbot$  OR TS="chatter bot$" OR TS="smart bot$" OR TS=smartbot$  OR TS=(Conversational NEAR/2 (assistant OR interface OR agent$ OR system OR computer OR bot$ OR AI)) OR TS=((virtual OR intelligent OR computer OR AI OR "artificial intelligence" OR embodied) NEAR/2 agent$) |  |
| 3 | TS=Aged OR TS=geriatric$ OR TS=elder* OR TS=senior$ OR TS=((old OR oldest$ OR older$) NEAR/2 (person$ OR adult$ OR individual$ OR people OR patient$)) |  |
| 4 | TS=((Mental OR Neurocogniti* OR Psych* OR Cogniti*) NEAR/2 (Disorder$ OR Illness* OR Disease$ OR Dysfunction OR Decline OR Deterioration OR Impairment$)) OR TS=(Memory adj2 (Disorder? OR Deficit OR Loss* OR Impairment?)) OR TS=Alzheimer$ OR TS=Schizophrenia$ OR TS=Parkinson$  OR TS=Huntington$ OR TS=Delirium OR TS=dementia$ OR TS=Amnesia$ OR TS=(Lewy NEAR/1 (Body OR Bodies)) |  |
| 5 | #1 AND #2 AND #3 AND #4 | 464 |
| 6 | #5 AND LD=(2024-04-24/2025-10-09) | 229 |
|  | **Total number of results** | 693 |

**Google Scholar (https://harzing.com/resources/publish-or-perish)**

**Date of the updated search:** 23-04-2024

**Database limit:** only up to the 20 first results per string have been considered; citation records and patents have been excluded.

| **#** | **Search** | **# Results screened** |
| --- | --- | --- |
| 1 | (participation OR involvement OR engagement) AND Digital AND ("old person" OR "old people" OR "old adult" OR "old patient") AND ("Mental Disorder" OR "cognitive Disorder" OR "cognitive Impairment" OR dementia OR Alzheimer) | 20 |
| 2 | (participation OR involvement OR engagement) AND Smartphone AND ("old person" OR "old people" OR "old adult" OR "old patient") AND ("Mental Disorder" OR "cognitive Disorder" OR "cognitive Impairment" OR dementia OR Alzheimer) | 20 |
| 3 | (participation OR involvement OR engagement) AND "mobile application" AND ("old person" OR "old people" OR "old adult" OR "old patient") AND ("Mental Disorder" OR "cognitive Disorder" OR "cognitive Impairment" OR dementia OR Alzheimer) | 20 |
| 4 | (participation OR involvement OR engagement) AND apps AND ("old person" OR "old people" OR "old adult" OR "old patient") AND ("Mental Disorder" OR "cognitive Disorder" OR "cognitive Impairment" OR dementia OR Alzheimer) | 20 |
|  | **Total number of results** | **80** |
|  |  |  |

**Google Scholar (https://harzing.com/resources/publish-or-perish)**

**Date of the updated search:** 09-10-2025

**Database limit:** only up to the 20 first results per string have been considered; citation records and patents have been excluded. Results have been limited to years 2024-2025.

| **#** | **Search** | **# Results screened** |
| --- | --- | --- |
| 1 | (participation OR involvement OR engagement) AND Digital AND ("old person" OR "old people" OR "old adult" OR "old patient") AND ("Mental Disorder" OR "cognitive Disorder" OR "cognitive Impairment" OR dementia OR Alzheimer) | 20 |
| 2 | (participation OR involvement OR engagement) AND Smartphone AND ("old person" OR "old people" OR "old adult" OR "old patient") AND ("Mental Disorder" OR "cognitive Disorder" OR "cognitive Impairment" OR dementia OR Alzheimer) | 20 |
| 3 | (participation OR involvement OR engagement) AND "mobile application" AND ("old person" OR "old people" OR "old adult" OR "old patient") AND ("Mental Disorder" OR "cognitive Disorder" OR "cognitive Impairment" OR dementia OR Alzheimer) | 20 |
| 4 | (participation OR involvement OR engagement) AND apps AND ("old person" OR "old people" OR "old adult" OR "old patient") AND ("Mental Disorder" OR "cognitive Disorder" OR "cognitive Impairment" OR dementia OR Alzheimer) | 20 |
|  | **Total number of results** | **80** |
|  |  |  |
